# Patients’ perceptions of use, needs, and preferences related to a telemedicine solution for HIV care in a Norwegian outpatient clinic: a qualitative study

**DOI:** 10.1186/s12913-024-10659-z

**Published:** 2024-02-15

**Authors:** Hege Mari Johnsen, Anita Øgård-Repål, Santiago Gil Martinez, Kim Fangen, Kristin Bårdsen Aas, Ellen Margrete Iveland Ersfjord

**Affiliations:** 1 Department of Health and Nursing Science, Faculty of Health and Sport Sciences, University of Agder, 4898 Grimstad, PO Box 509, Norway; 2 Sørlandet Hospital, Kristiansand, Norway

**Keywords:** Digital health services, Digital pathway, Human immunodeficiency virus, Person-centred care, Shared decision making, Telehealth, Vulnerable groups

## Abstract

**Background:**

Telemedicine in outpatient services for people living with human immunodeficiency virus (PLHIV) was scaled up during the COVID-19 pandemic as services transitioned to remote care. Many studies have reported on the challenges and advantages of telemedicine care during the pandemic. However, there is limited research on the provision of telemedicine human immunodeficiency virus (HIV) care beyond the COVID-19 pandemic, which entails different telemedicine components and focuses on ways to improve the telemedicine experience for patients. This study aimed to explore PLHIV’s perceptions of use, needs, and preferences related to a telemedicine solution for HIV care in an outpatient clinic in Norway. The telemedicine solution included a pre-consultation questionnaire, asynchronous digital messages, and video consultation.

**Methods:**

Qualitative interviews were conducted with 12 PLHIV. The interviews were analysed using thematic analysis.

**Results:**

We identified four main themes that covered the participants’ perceptions, needs, and preferences: (1) perceived usability, (2) maintaining confidentiality, (3) accommodating personal preferences, and (4) perceived usefulness. Some participants had difficulty logging into the telemedicine solution. Other participants suggested additional functionalities, such as picture sharing and access to test result. Telemedicine care enabled the avoidance of stigmatising clinic experiences, although a few participants reported concerns about confidentiality and data security. Accommodating personal preferences and needs in terms of the type of consultations (in-person or video) and frequency of visits was essential to the participants. With telemedicine care, participants felt more in control of their own lives, perceiving that it increased their perceived quality of life and saved them both time and money for travelling to the clinic.

**Conclusions:**

Our study identified several specific needs and preferences related to the assessed technical solution and the provision of current and future telemedicine care services. Nevertheless, the telemedicine solution was perceived as a usable, flexible, and person-centred approach to HIV care, contributing to accommodating the participants’ personal preferences. However, healthcare professionals need to ensure that individual requirements and preferences are consistent with evidence-based follow-up and supported by person-centred care. Thus, the practice of shared decision making is important in telemedicine care.

**Supplementary Information:**

The online version contains supplementary material available at 10.1186/s12913-024-10659-z.

## Background

Due to advances in antiretroviral therapy of the human immunodeficiency virus (HIV), well-treated people (virally suppressed or undetectable viral load) living with HIV (PLHIV) do not pass the virus on [1, 2]. Thus, HIV has become more like a lifelong chronic disease for PLHIV receiving treatment. As PLHIV age, they are at a higher risk of developing co-morbid conditions at a younger age than individuals who are HIV negative [1, 3, 4]. In addition, the health-related quality of life of people with HIV is lower than in the general population. This is explained by factors unique to HIV infection, such as the communicable character of the disease and isolation from society due to stigma and discrimination [5, 6]. Hence, PLHIV need follow-up related to their HIV diagnosis, possible co-morbid conditions, and emotional health and well-being.

An individual’s ability to self-manage a life with a chronic disease (such as HIV) has been shown to improve patient outcomes and quality of life [7, 8]. Thus, telemedicine (connecting with healthcare professionals online) has become an important tool to succeed with patient-centred health services and the development of a sustainable healthcare service for the future [9, 10]. Telemedicine care may include talking to healthcare professionals on the phone or video chat, sending and receiving messages, and the use of remote monitoring using a computer, tablet, or smartphone [11]. Similar to other outpatient clinic (OPC) services, HIV OPCs have transformed their services by incorporating telemedicine care to complement existing services. This evolution has happened in response to the changing needs to individualise and improve the quality of treatment and care and to increase patients’ self-management of their disease [8, 12]. Internationally, telemedicine in OPC services for PLHIV was scaled up due to COVID-19 [13–15]. The use of telephone visits was more common than video visits during the pandemic. This was partly explained by video access, technological literacy, and resource limitations [14, 15]. The identified benefits of using telemedicine for HIV treatment among PLHIV were convenience, avoiding stigmatising clinic experiences, perceived patient-centredness, scheduling flexibility, and saving of time and transportation costs for patients living far distances from clinics [13, 15–17]. Identified barriers include privacy and confidentiality (data security) concerns, such as a lack of conducive environments and private spaces to speak with healthcare professionals remotely, and concerns that information could be recorded and circulated without one’s control. Additional barriers include technology literacy and technical challenges and the loss of routine clinical experiences and interactions [13–15, 17]. Barriers are often related to a lack of a household, lower income levels, lack of technological resources, or other languages [15].

There is a need for more studies focusing on the provision of HIV care using telemedicine beyond the COVID-19 pandemic and on ways to improve patients’ telemedicine experience [15, 18]. Specifically, there is a lack of research assessing patient preferences and needs for telemedicine or in-person visits to improve acceptability and continuity of care [15, 16]. In addition, given that video and telephone visits seem to be the most frequently reported modalities, research is needed on the experiences of using other telemedicine technologies [17]. Thus, the aim of our study was to explore PLHIV’s perceptions of use, needs, and preferences related to a telemedicine solution for HIV care in an OPC in Norway. The telemedicine care solution entailed the following components: a pre-consultation questionnaire, asynchronous digital messages, and video consultation. The main entry point to the telemedicine solution was the national health network in Norway (helsenorge.no). The prototype of the telemedicine solution was tested at the HIV OPC in 2021–2023. This telemedicine care solution was initiated by PLHIV and developed in collaboration with patient representatives, health, and ICT personnel. This work was planned prior to and carried out despite the COVID-19 pandemic. The OPC’s aim of implementing telemedicine care was to enable patient follow-up beyond in-person meetings, and thereby be a supplement and even an extension of the OPC’s existing services.

## Methods

### Study design

A qualitative design was utilised [19] to explore patients’ perceptions of use, needs, and preferences related to a telemedicine solution for HIV care in an OPC. Individual interviews were conducted with 12 PLHIV, who received follow-up and treatment at an HIV OPC in Southern Norway. Data collection was conducted from March to June 2022.

### Context

An organisational redesign of the HIV OPC was conducted in 2012 [20], with the aim of achieving optimal health, holistic care and treatment, and empowerment of the patients. The organisational redesign entailed establishing a user board, a partly user-driven OPC for PLHIV, and a position for an HIV nurse coordinator at the OPC. Among a set of user-defined targets for the follow-up of PLHIV made by the user board were implementing telemedicine care for outpatient follow-up. The need for digital communication during the COVID-19 pandemic accelerated the initiative for the development and pilot testing of a prototype of telemedicine care at the HIV OPC from August 2021. Since there was no funding for developing new technology, already existing technical software for a pre-consultation questionnaire and video consultation were added to the content in the national health network platform “helsenorge.no”, an encrypted secured platform for the communication of health data. The asynchronous digital message service was an included component of the national health network platform.

### Participants

The participants were recruited by the HIV coordinator (KBA) at the HIV OPC. A strategic sampling method was conducted based on the following inclusion criteria: the patients (1) were already enrolled as service users at the OPC, (2) were considered well treated (the viral load was under control) and had a generally stable health condition, (3) had basic knowledge about living with HIV, and (4) were motivated to try telemedicine care. In addition, the patients were considered suitable to be followed up digitally by the HIV coordinator in collaboration with a physician. The patients received information about the telemedicine solution and related study from the OPC nurse (KBA). If they volunteered to participate in the study, they received more information about the study from the patient consultant (KF), which was based on an information letter from the researchers. To contact the participants, the first author (HMJ) received 15 names and phone numbers from the patient consultant by phone. The provision of contact information was conducted in accordance with the hospital’s own guidelines and regulations. Based on their preferences, the participants were contacted by HMJ through short message services (SMS) or called up. HMJ did not know any of the participants in advance. Two participants did not reply to the first or second SMS. One participant actively withdrew from the study. In total, 12 PLHIV participated in the study.

### Data collection

The data were collected through semi-structured interviews. The interview guide was developed for this study and contained questions about participants’ background, experiences with the follow-up prior to the introduction of the telemedicine care solution, perception of using the piloted telemedicine solution, and needs and expectations regarding the solution and the implementation of this service (Additional file 1). The questions were reviewed and quality assured by a patient consultant and a senior medical physician at the OPC.

Information about the study was communicated both written (from the OPC) and by phone (by HMJ). This included information about the study aims, their right to withdraw, ethical processing and preservation of data, and reporting of the results. Informed oral consent was obtained from all participants before data collection began.

All the participants chose whether the interview should be conducted digitally (MS Teams or Zoom), by phone, or in person. Six interviews were conducted by phone, and four were conducted by MS Teams or Zoom. Two interviews were conducted on a university campus and at a café. Most of the interviews were conducted by two of the authors (HMJ and EMIE). However, due to some participants’ desire to carry out the interviews straight away when they were contacted by SMS or phone, five of the interviews were conducted only by HMJ. The interviews were recorded with a separate offline audio recorder. The interviews were conducted from April to June 2022 and lasted between 18 min and 1 h, with an average of 40 min.

### Analysis

In this study, we employed an inductive and data-driven analysis approach based on Braun and Clarke’s thematic analysis [21]. First, HMJ, AØR, and EMIE read the transcripts and familiarised themselves with the data. Second, the transcription task was divided among the three authors and initially coded individually. Next, the three authors met to revise the coding, cluster codes, determine evident patterns, and generate initial themes and subthemes. Finally, the initial themes and subthemes were reviewed and discussed by all authors to expand understanding, resolve possible conflicts of understanding, and reach consensus on final themes and subthemes. Table 1 shows an example of the analysis process.

**Table 1:** Example of the thematic analysis process

Participant statement	Code	Sub-theme	Themes
“*I began to struggle with sleep. It had nothing to do with the follow-up, but it had something to do with the fact that I had to go into the hospital and maybe meet someone I knew, those who worked in that corridor*”.	Fear of being seen at the HIV clinic	The physical space	Confidentiality

In the result section, we present direct quotations from the interviews (translated from Norwegian into English). To ensure the anonymity of the participants, no information about each participant or their number is displayed in presenting the results. The reported prevalence of participants’ responses is generally presented using descriptors such as “most”, “many”, and other equivalent terms. The manuscript preparation adhered to the COnsolidated criteria for REporting Qualitative Research (COREQ) checklist.

## Results

The participants comprised 1 female and 11 males. Their ages ranged from 26 to 70 years (median: 45). The time since HIV diagnosis ranged from 2 to 20 years (median: 8 years). Nine of the participants had a consultation once a year, while the other three participants had one consultation every six months. Five participants were in a relationship, and seven were single or divorced. Nine of the participants were employed and/or students. Three participants were receiving disability pension or retired. Five of the participants had started their treatment for HIV in another country or another Norwegian county other than the current OPC. Self-reported competence in using a computer varied among the participants, from limited to high.

Through the analysis, we developed four main themes: (1) perceived usability, (2) maintaining confidentiality, (3) accommodating personal preferences, and (4) perceived usefulness. Each theme included different aspects that were sorted into subthemes. The main themes and subthemes are displayed in Table 2.

**Table 2:** Main themes and subthemes

Main themes	Subthemes
Perceived usability	Access Functionality
Maintaining confidentiality	The physical space The digital space
Accommodating personal preferences	In-person consultations Need for flexibility
Perceived usefulness	Perceived impact Prerequisites for a positive impact

### Perceived usability

This theme covers participants’ perceptions related to the usability of the telemedicine solution, which entailed the following components: a pre-consultation questionnaire, asynchronous digital messages, and video consultation.

#### Access

Most participants found it easy to access (via a log-in functionality) the national health network (Helsenorge.no), where they gained access to the three separate technical components (pre-consultation questionnaire, asynchronous digital messages and video consultation). This was explained by the fact that this platform was already used to receive other correspondences regarding treatment, view the results of COVID tests, and schedule consultations and/or order prescriptions from their general practitioners (GPs). Therefore, using helsenorge.no to access the telemedicine solution was perceived as an advantage. This was partly explained by the fact that this was a national health network, and that a user had to use a unique identifier (in Norwegian, bank ID) to get access.

Many participants experienced difficulty finding links to access the pre-consultation questionnaire and the video consultation. These links were placed in two of the written correspondences from the OPC regarding the telemedicine solution and the associated pilot study. The links were placed between large sections of text and were perceived as challenging to find. One participant reported:

I wish it [the information letter] was clearer, like “Here is the link to the [pre-consultation] form, here is the link to the video meeting.” Because now, I felt like I had to search for them. So, it was a bit cumbersome to find my way around.

As one participant expressed it, “*The content of the letters was messy*”. Some of the participants had called the nurse in the OPC for guidance on accessing the solution. Other participants had overlooked information in the first correspondence about entering a link to agree to testing the telemedicine solution. They were contacted by the nurse at the OPC, who made them aware that they needed to approve the digital agreement before telemedicine care could start. One of the participants suggested that finding the links should be made much more straightforward, particularly for those users who had limited competence in using a computer, such as the participant. He/she suggested that there should be available guidelines on learning how to use the telemedicine solution, because it was easy to forget how it worked the first time due to the amount of time lapsed until the next consultation (i.e. many PLHIV have their consultation only once a year).

All the participants expressed that during their initial navigation of the provided links, accessing the three separate solution components was easy. However, the video consultation required that the physician or nurse enter a code to start the video consultation. Some participants were unsure whether they also needed to add a code to gain access. One participant explained:

There was this thing that popped up asking for a PIN code. And then, I went back to my emails to find information about the digital system, but there was nothing mentioned about a PIN code. So, I just logged in, and I was able to access it without entering a PIN. So, it was actually a question that was completely unnecessary.

None of the participants complained that the telemedicine solution required the use of different technical components. However, one participant complained about the multiple log-ins: “*It’s always silly to have several different log-ins or several different places you have to log-in*”. The participant proposed:

Preferably, the three separate technical components should have been able to access directly from the national health network and be visible in the website’s menu, like other services on the same platform, instead of having to look for links in the [information] letter.

#### Functionality

The participants appreciated the possibility of communicating with the OPC healthcare professionals through electronic messaging and video conference. All the participants perceived using digital messages as easy: “*There was a reply button, and you wrote what you wanted to write and then you got a reply back. So, it was really alright*”. However, one participant wished for the opportunity to send a direct message to either the nurse or the physician:

After the video call, you should have the option to send direct messages solely to the person you had the conversation with, and you know that only they can see it, then you can follow up with anything you may have forgotten during the conversation.

Almost all participants did not experience any technical issues with the sound or picture during the video consultation. One participant experienced that his/her screen was unresponsive but acknowledged that it could have been due to problems related to internet connectivity. Another participant was unsuccessful when trying to get the video in a full-screen view while using a mobile phone for the video consultation, and wondered whether this was possible. Similarly, another participant mentioned that the pre-consultation questionnaire could probably be easier to complete using a computer. Most of the participants used their phones for telemedicine care. However, one participant explained that this was due to the lack of a web camera on his/her computer.

Most participants found the functionality of sending information about their status before attending their consultation using the pre-consultations questionnaire useful. One participant pointed out that it was good that the pre-consultation questionnaire included both physical and psychological subjects, because it does not always feel natural to bring these subjects up in the consultation. All the participants appreciated the possibility of writing comments on the questionnaire. They also found being able to request to talk to a peer useful.

The digital pre-consultation questionnaire was perceived by some of the participants as too long and general. For example, one participant outlined an important drawback;

One had to answer all questions, including irrelevant ones, without the option to mark them as not applicable. By removing irrelevant items, the questionnaire could have been more personalized.

Other participants desired the possibility of grading their reply instead of replying “yes” or “no”. For example, one participant suggested that when a user had the same problems as last time, it should be possible to respond whether they were better or worse, or how much it bothered them. In addition, they indicated that the questionnaire should have asked more than whether the user had any new physical or psychological problems. One participant felt it was difficult to remember and fill in some of the answers, as one year had passed since the last consultation. Only one participant had experienced that the consultant physician did not have access to his/her pre-consultation questionnaire.

One of the participants expressed that he/she desired the functionality of sending pictures digitally, as he/she experienced many skin problems that he/she assumed were related to his/her HIV treatment. A few of the participants also wished to view the results from their blood samples. However, one of the participants stated that he would not have understood the values anyway and had no need to view them. Similarly, another participant thought such values could cause concern if the patient did not have adequate knowledge: “*I would have gotten a little… more scared if I just got an answer without someone being there to tell me that it went well*”.

When they were asked whether the solution should include the possibility of communication with an HIV peer, the participants’ opinions varied. Some thought this was a good idea. Others had no need or were sceptical due to the possibility of receiving wrong advice related to their HIV treatment, even though peers were officially trained and approved by the hospital.

It was proposed by the participants that the healthcare professionals at the OPC should assess whether the patients offered telemedicine care had the necessary digital equity (i.e. digital competence and equipment) for this type of follow-up. Most importantly, “*It should be as simple as possible. Then as many as possible can use the telemedicine solution*”.

### Maintaining confidentiality

This theme covers participants’ needs and expectations related to maintaining confidentiality in HIV care and how these are met in physical and digital appointments.

#### The physical space

Most of the participants had not told anyone other than their close family about their HIV diagnosis. This was due to the fear of facing stigma and discrimination from society. One participant expressed, “*If you have zero virus, then you cannot infect anyone. But very few people know that, even nurses and physicians*”. Due to the fear of societal prejudice and stigma, confidentiality was perceived as important to the participants. Hence, several participants expressed that being physically at a hospital felt uncomfortable, especially in the waiting room in the OPC. One participant expressed it like this:

The only thing I remember is that I found it very uncomfortable to go to the hospital. Yeah, that’s actually what I still feel a bit ashamed about. What I don’t like is that you sit in the hallway. And in the beginning, you’re so uncertain, you’re in a state of shock and you’re scared.

Feeling uncomfortable was particularly mentioned as a problem by participants who had positions in the healthcare sector or knew someone who worked at the hospital. For instance, a few of the participants had chosen to have their follow-up at the OPC instead of at their local hospital to prevent meeting people they knew.

However, some of the long-term PLHIV proposed that the feeling of discomfort had decreased as current waiting rooms were no longer diagnosis-specific. Due to the need for confidentiality, most of the participants were satisfied with having in-person visits at the OPC only once or twice a year.

#### The digital space

The ability to avoid physical appointments through digital consultations was perceived as a major advantage among all participants. Other advantages pointed out were receiving information about their appointment in a digital mailbox instead of receiving a letter at their home address, receiving an SMS alert on their mobile phone when they have messages or documents in the national health portal, and the ability to send digital messages instead of giving their name and personal messages to the secretary at the OPC to be forwarded to the nurse. Despite these advantages, the participants also pointed out new challenges in relation to telemedicine care and confidentiality. For example, two participants were sceptical about information security. One of them said, “*I’m also a little uncertain about who reads the digital messages. So, I didn’t want to answer that much, […] I would rather talk about certain stuff over the phone*”. In addition to the concern about not knowing who read their information, there was also mistrust in the Norwegian healthcare system’s handling of personal data in general. For example, one participant who worked at a hospital said:” *In the beginning, when I got the diagnosis, I went in and checked who had been looking at my medical record. It’s so silly, but that’s just how it is”*. Despite being sceptical about confidentiality, one participant mentioned a positive experience he had related to data security:

The doctor who is my regular doctor was sick on the day of my scheduled consultation. So, he [the substitute doctor] couldn’t access the digital form due to security measures. I found that to be very reassuring. But it was also a bit inconvenient, because I didn’t remember what I had answered on the questions.

All the participants preferred to have video consultations with healthcare professionals they already had met in person at the OPC. They also mentioned that the environment in which they conducted their consultation could become a challenge in regards to confidentiality: “*If you don’t have earplugs, anyone can hear the sound. So don’t start the conversation when you’re sitting in a café* [laughter]”. Similarly, the participants suggested avoiding digital consultations through hands-free devices in a non-moving car. In addition, none of the participants had disclosed to their employer or colleagues about having HIV and proposed that it could be a challenge to have a video consultation at the office during working hours. It was also suggested that it could be a challenge to have a digital consultation at home when not all your family members knew about your diagnosis.

Two of the participants pointed out that a negative thing about changing from in-person to digital consultations could be that the group of HIV patients is further hidden from society, which may reinforce stigma.

### Accommodating personal preferences

This theme covers participants’ needs and expectations related to receiving telemedicine HIV care.

#### In-person consultations

All the participants suggested that it should be up to the individual patient to decide when the use of telemedicine could start. As one of the participants put it,

I think telemedicine is great for all of us who have figured things out, who don’t struggle with anxiety and mental issues as a result, or who worry. I don’t think it’s suitable in an early stage […] For early patients, I absolutely feel that one must still have a lot of contact with the physician and nurse.

Similarly, all the participants expressed that when people are diagnosed with HIV, it is essential that the first consultations at the OPC are in person, until the patient has received enough information and medical treatment follow-up and feels safe about handling their new life situation. One of the participants pointed out the importance of initial in-person consultations.

I think I’m the luckiest patient in the world, quite simply […] I went from sitting at home sorting papers and planning to end my life to suddenly, within 3 months, handling everything and looking forward to things. It’s not bad that they [healthcare professionals] were able to achieve this in such a short time. I think I’ve been very lucky with them (OPC).

The need for in-person visits was also highlighted by others who had less frequent consultations. For example, one participant expressed that “*It is good to be able to meet in person now and then if you feel for a hug or a stroke on your arm by the OPC nurse*”.

#### Need for flexibility

Most of the participants agreed that the frequency of consultations (3, 6, and 12 months) was adequate. However, some participants proposed that the need and frequency of consultations could change depending on their life situation. For example, two participants wished to be contacted by the OPC nurse in between regular yearly appointments to prevent the feeling of discontinuity of their care. Some indicated that it felt good to talk to someone and to feel taken care of, even if there were no particular health issues at that moment. Only one of the participants expressed that every 12 months felt too frequent: “*She [the nurse] tells me you are healthy, there is no change and everything is going well, So it’s kind of a waste of my time*”. This person would prefer receiving an e-mail or digital message telling him/her that the blood samples looked within the safe interval.

One of the participants, who lived in another region, had already used a flexible form of follow-up by having some of the consultations by phone. He stated, “*If I had to travel every 6 months to the OPC, it would have been really impractical*”. Other participants had work that required a substantial amount of travelling, which was experienced as inconvenient when they had to go to physical consultations. For example, one patient who worked abroad faced difficulties in countries that did not grant entry to PLHIV. The ability of digital consultations would make consultations much easier and more flexible. Being able to send messages after working hours was also perceived as an advantage. Others also appreciated this flexibility: “*The more choices you get, the easier it is because no patients are the same*”. The participants recognised that the ability to send digital messages to the OPC nurse could meet the need for flexibility in their HIV follow-up. This was explained by the fact that, “*Usually, there are just very simple questions that you are wondering about*”. The flexibility of sending messages was also viewed as a challenge for some participants: “*I’m not entirely comfortable and sure yet how quickly they read it when I send something*”. Some of the participants had experienced that it took some time before the nurse answered their messages (e-mailed or called back).

Most of the participants expressed that telemedicine care should be a supplement rather than a full replacement for OPC consultations. Additionally, several of the participants wished they could have their blood samples taken at their GP’s office to avoid going to the hospital only for the blood samples. Currently, most patients need to take blood samples one or two weeks prior to their scheduled appointment at the hospital. One participant expressed that his/her therapist at the OPC had explained that they seldom give GPs access or responsibility related to the treatment of HIV, because this requires education about HIV in particular.

### Perceived usefulness

This theme covers participants’ perceived impact of telemedicine HIV care on their own and other patients’ lives, and important prerequisites for a positive impact of this kind of follow-up.

#### Perceived impact

Several participants expressed that the ability to use telemedicine had a positive impact on their perceived quality of life:

I think it’s absolutely great. It makes you feel healthier; it makes you not have to go to a hospital. It is much more effective in everyday life. Letting go of taking time off work to sit for a whole day and […] or for me then, I have to fly down to the OPC. So, it is really an improvement compared to earlier.

Similarly, one participant expressed that, “*With telemedicine care you get to have more control over your own life, and it’s also good for your mental health*”. According to one participant, going to the OPC affects one’s own self-esteem: “*When you’re in the hospital, that’s when you feel sick*”. Further, many participants pointed out that telemedicine offers more opportunities for personalised follow-up:

Some people only need the one contact a year and get information about the blood samples and that everything is fine and stable, take the medicines, and just go on with their lives. And I notice that I also become more like this as time goes on. But then there are others who struggle with things, other diseases, and complications that come from this disease. So, then you need closer follow-up, and you may need to talk to a peer and have more contact with a nurse or doctor.

According to several of the participants, the ability of digital consultations not only made consultations easier and more flexible but also saved both time and money for travelling to the hospital, particularly those who travelled from other regions in the country for their scheduled consultation. They also stated that saving money could be for the better of others: “*Clearly, if you can cut the costs for all of us who don’t really have the needs (for extra follow-up), then that money can be used on the patients who need more than what they actually receive*”.

#### Prerequisites for a positive impact

Many participants expressed that the most important aspect was seeing the individual patient, whether digitally or in person. As one put it, “*I expect to be met in the same way as when you meet in person*”. Furthermore, a positive experience with digital consultations was also linked to the personality of the person you met during the consultation:

It is not the technical solutions in itself that you really need […] I would say that it is rather up to the staff that you get good enough physicians who actually have time to take care of people. Because it’s clear that if I hadn’t had the physician that I had (at the OPC), things would have been much more difficult. I know that because I have come across others who are not so good and are neither updated nor able to see what you actually need.

Some participants also pointed out that it was important to perceive that OPC personnel were informed and prepared before their consultation. One of the participants proposed that their responses in the digital questionnaire should be used actively by OPC personnel during the consultation.

## Discussion

The aim of this study was to explore PLHIV’s perceptions of use, needs, and preferences related to a telemedicine care solution for HIV care in an OPC in Norway. The solution included the following components: a pre-consultation questionnaire, asynchronous digital messages, and video consultation. The study was conducted in collaboration with the OPC with the intention of evaluating and possibly improving PLHIV’s telemedicine experience. The findings of this study are discussed according to the main themes.

### Perceived usability

Scholars have recommended that healthcare organisations adopt and implement telemedicine platforms that are user-friendly and easy to navigate [17, 22]. This is because technical challenges can negatively impact appointment flow, intervention effectiveness, and the satisfaction of both the patients and healthcare professionals involved [22]. When introducing telemedicine care at the OPC in this study, the hospital trust found it sustainable and more affordable to reuse existing software for the pre-consultation questionnaire and the video consultation. Consequently, participants had to use separate links embedded in documents received through the national health network platform to access the pre-consultation questionnaire and the video consultation. The participants experienced difficulties finding the embedded links to access these two components and had to receive guidance from the nurse. Based on our findings, access to the solution should be made more seamless and include user support. Furthermore, the present pre-consultation questionnaire should preferably be revised in collaboration with PLHIV to capture their perspectives on what questions are relevant and can easily be completed [23].

The participants also expressed a need for additional functionalities, such as the ability to direct their digital messages to a nurse or physician, send pictures, and view blood sample results. Previous studies have recommended that telemedicine solutions meet users’ needs and preferences [15, 16, 24]. However, all the proposed functionalities are not necessarily appropriate, as usefulness depends on the user’s circumstances. For example, when digital messages are sent to a nurse or physician, there is a risk that the message will not be read if that person has time off or is absent for other reasons. In addition, as proposed by one participant, patients may be upset when they read the results from their blood samples but are not able to talk to healthcare professionals.

The participants required comprehensive and available instructions about how to access and use the different components. This finding supports previous research showing that technical literacy is a critical factor in sustaining telemedicine for HIV care [13, 14, 17]. Even if the OPC had provided the participants with information about the telemedicine solution and its use prior to telemedicine care, the patients could easily forget such information, especially since they only used it once a year (as suggested by one participant). Based on our findings, the OPC has already started improving its information material. For example, they developed both written and digital information and made short videos about telemedicine for HIV care.

In addition, one participant proposed that the healthcare professionals at the OPC should have assessed whether the PLHIV who were offered telemedicine care had the required digital equity for this type of follow-up. This is in line with recommendations by Labisi et al. [17], who proposed that healthcare professionals should consider incorporating checklists to ensure that patients can navigate included telemedicine platforms before proceeding with the use of this kind of HIV care.

Research has shown disparities in access to telemedicine in HIV care [13, 14, 17]. For example, technological barriers include a lack of broadband access in rural regions, a lack of compatible technological devices, and a digital divide gap in minority populations. All our participants had access to a computer and a telephone and generally had a stable internet connection. Only two of our participants lacked equipment, such as a computer and/or telephone headset. None of the participants reported barriers to access to telemedicine related to low-income levels or a lack of a household. Therefore, the participants appeared to be more resourceful than the PLHIV included in research from other countries. One explanation for this is that Norway has a very high standard of living compared to other European countries. The country also has one of the world’s best health and welfare systems, providing housing, life support, and necessary equipment for people with limited resources [25], as well as a highly developed internet infrastructure. However, the aforementioned potential barriers should be taken into account when broadening the inclusion of PLHIV in telemedicine care.

The fact that a telemedicine solution complies with usability heuristics does not necessarily mean that a patient’s overall experience is positive [16]. Therefore, it is important to understand the individual experience when using digital solutions (such as our telemedicine solution) and how their design affects them. In addition, Davis et al. [26] proposed the triangulation of usability methods for eHealth HIV interventions, which is on the agenda of the authors.

### Maintaining confidentiality

Consistent with participants from other studies [17, 24, 27], most of the participants in our study were concerned about stigmatising clinic experiences and being seen at HIV clinics. However, researchers have found high levels of satisfaction reported by PLHIV as telemedicine users, especially because they could avoid coming to the clinic [17, 27]. For example, patients who had telemedicine visits were less likely to miss appointments than those who had in-person visits. Thus, to prevent PLHIV from cancelling or not showing up to appointments due to stigma, telemedicine may be an alternative to in-person visits when patients do not require laboratory work. However, as two of our participants pointed out, the stigma does not go away by “hiding” PLHIV from society through telemedicine care. Living with an HIV diagnosis, which is so closely linked to stigma, means that even when telemedicine solutions are considered safe, the fear of disclosure may prevail. The literature shows that HIV-related stigma may have severe consequences for PLHIV’s health, as stigma influences healthcare behaviours and prevents PLHIV from accessing healthcare services and treatment [5, 6]. Hence, there are calls for action by governments to raise public awareness and develop education campaigns about HIV for healthcare professionals and the public to decrease the stigma related to PLHIV [28].

Concerning confidentiality, the participants perceived that it was a great advantage that all communication with the OPC could now be digital, which meant that they avoided receiving appointments or other documents from the OPC in their home mailbox. However, some participants were concerned about confidentiality related to others’ access to read their digital messages and the lack of conducive environments and private spaces to speak with providers remotely. Similar concerns about information security and privacy have been identified in other research related to the use of telemedicine in HIV care [14, 15, 17]. An explanation for the concern about data security can be that various software packages are used in healthcare delivery. Some health organisations have their own software, while others consult third parties [17]. Privacy and data security considerations need to be recognised at all stages of the development and implementation of telemedicine solutions [29]. Regardless of the type of software package used for telemedicine, Labisi et al. [17] and Wotton et al. [22] recommended that a regulatory government agency oversee these platforms and ensure that they meet specific requirements. This is essential for earning patients’ trust in telemedicine and reassuring them of protected data. In line with these recommendations, Norway has strict legislation and policies for national e-health services and ICT infrastructure.

The national health network in Norway was used to deliver the telemedicine solution in our study. The digital message service, video consultation service, and pre-consultation questionnaire were all secured and nationally approved components. However, all digital messages were received through the patients’ electronic health records (EHR) and were subsequently sorted by the OPC secretary and directed to either the patients’ nurse or physician. This means that all healthcare professionals who had access to a particular EHR working group could view the patients’ messages. Based on our participants’ concerns about confidentiality, patients should be better informed about who reads their messages, what content is appropriate for the digital message service, and whether sensitive information should be shared by telephone or during in-person or video consultations. In contrast to the digital messages, the pre-consultation questionnaire and the video consultation were separate components outside the EHR that required the OPC nurse and physician to log in with separate passwords. As mentioned by participants, this secured confidentiality but could cause trouble if substitute healthcare professionals did not have access to all the components included in the telemedicine HIV care solution. Hence, the OPC needs to ensure necessary access for its personnel to prevent challenges to in-patient consultations.

### Accommodating personal preferences

All the participants agreed that the ability to send digital messages and have video consultations would make their follow-up much easier and more flexible. It was perceived as particularly convenient for those who travelled very frequently or lived far from the OPC. However, the need for in-person visits and preferences for the frequency of visits seemed to vary among the participants and could change depending on their life situation. In these kinds of situations, or when in need of a physical examination, telemedicine cannot replace human contact [14, 30]. Further, our participants expressed that in-person visits for newly diagnosed individuals are essential and that transition to telemedicine visits only should occur if and when patients feel safe and comfortable with this change in care. This has also been expressed by participants in other studies [13], and is in line with the proposition of Mgbako et al. [24] that telemedicine should first serve the patient’s needs and be person-centred. By giving PLHIV the autonomy to choose their preferred mode of delivery for each visit, healthcare organisations can bolster HIV self-management and improve attendance rates [17, 24].

Shared decision making is the cornerstone of patient-centred care, where healthcare professionals and patients work together to reach a shared and appropriate choice of healthcare [31]. For example, one participant considered a medical consultation that occurred once a year to be “too frequent”. This shows that there is a need for evidence-based knowledge and information from the physician to explain why in-person visits are necessary for the patient’s condition. Furthermore, the frequency of in-person visits (for treatment and follow-up) may need to accommodate patients’ perspectives and preferences [31]. On the other hand, some participants expressed a preference for more frequent consultations than those scheduled. Since the OPC has specific criteria and resources for the provision of HIV care, there are limitations to the number of consultations available. In addition, the need for consultation is not necessarily related to medical issues. Thus, a shared decision making process can facilitate uncovering other needs, opening the door to the participation of other professionals in such a process (i.e., social workers or mental health specialists). According to Mgbako et al. [24], a team-based care approach will need to be incorporated into the telemedicine model, as care for PLHIV is typically performed, in most cases, by a team of physicians, nurses, mental health specialists, social workers, and care coordinators.

Our findings align with other research showing that telemedicine is perceived as a flexible approach to care and that a combination of in-person care and telemedicine may increase overall access to HIV care [13, 14].

### Perceived usefulness

Our participants perceived that the use of telemedicine gave them more control over their own lives and increased their quality of life. For instance, the feeling of control included not having to travel to the OPC. Reported benefits included less stress, a better fit within work schedules, and saving time and money for transportation, which correlate with the benefits identified in other studies [13, 15–17]. Therefore, it appears that the OPC has succeeded in building a telemedicine model in HIV care that increases the patient’s degree of independence and self-management [30], and that empowers the patient [24]. In addition, as confirmed through our findings, the flexibility offered by telemedicine HIV care has improved the OPC’s ability to provide person-centred care and improve patient health outcomes [13]. However, our participants’ perceptions and preferences of telemedicine care also support the fact that a patient-centred approach to HIV care also depends on trust, a good patient–provider connection, and effective communication [24]. For instance, it was important for the participants to have met healthcare professionals in person before starting telemedicine care, which could familiarise them with their face and body language. In line with the findings of other research [22, 27], differences in how healthcare professionals appear to the patient over video-conferencing (e.g. tone, volume, and expression of emotion) may negatively affect how a patient experiences a consultation. Our participants also emphasised the importance of a well-prepared healthcare professional—that is, one who actively used the information in the completed pre-consultation questionnaire during their consultation. The prerequisites of positive impacts or experiences perceived by our participants are in line with those reported by Baim-Lance et al. [14], who showed that patient–provider interaction during telemedicine was according to expectations, particularly when patients already had an established relationship with their providers, helping to achieve their communication goals. To provide a patient-centred approach, healthcare professionals should continually appraise their telemedicine programmes through patient feedback and consider provider education training on optimal communication to enhance trust and connection with the patient [24].

### Strengths and limitations

Values of trustworthiness, such as credibility, dependability, and transferability [32], were protected by the procedure we chose for conducting our qualitative study. The criteria for credibility may be understood as keeping the focus of the project. This was achieved by appropriately matching the methods to empirical questions and issues and choosing participants according to pre-defined criteria by experts in the field of HIV care and patient experience– all of the aforementioned relevant to the aim of this study. Dependability was achieved by having the same two authors (HMJ and EMIE) conduct the interviews and using the same interview guide for all participants. The main author (HMJ) contributed to all the interviews. HMJ is an intensive care nurse and associate professor and has expertise in health informatics research and education. The other interviewer (EMIE) is a medical anthropologist, associate professor, and postdoctoral research fellow in e-health. She contributed to eight interviews.

In relation to the credibility of the data, the three authors had experience with qualitative research and conducted the data analysis. The researcher (AØR) who participated in the data analysis together with HMJ and EMIE is a mental health nurse with a PhD related to peer support at outpatient clinics for PLHIV. In addition, all four authors—HMJ, EMIE, AØR, and SGM—have extensive research experience within the field of patients’ experiences of health services and new treatment methods, including e-health. In relation to transferability, we have described the context, the participants, and the research process thoroughly. It should be possible to achieve the same standards and outcomes in other similar studies. Therefore, we argue that our qualitative study meets the criteria for trustworthiness.

Our study has some limitations. First, the participants were recruited by two of the authors—one works as a nurse (KBA) and the other is a patient consultant (KF) at the OPC. Recruitment was conducted in accordance with relevant guidelines and regulations to avoid putting any pressure on the participants to contribute to the study. In addition, the two authors (KBA and KF) did not read any transcripts of the data to maintain the anonymity of the participants. They only contributed to the interpretation and discussion of the results, and the reading and approval of the manuscript. Second, the transcripts were not returned to the participants for comments and/or corrections. However, the participants were invited to a planned workshop in which the goal was to discuss the results and agree on the provision of future telemedicine care with the personnel at the OPC and ICT personnel. Third, the sample size was limited. However, the data collected from the 12 participants reached a point where no new information was discovered, a state called data saturation [19].

## Conclusion

The telemedicine care solution used in this study was initiated by PLHIV and developed in collaboration with patient representatives, health, and ICT personnel. It was piloted in an OPC in Norway from 2021 to 2023. In this study, we explored PLHIV’s perceptions of use, needs, and preferences related to use of this telemedicine solution. The findings in this study will inform future telemedicine care for PLHIV and other groups of patients who receive follow-up at an OPC at the same hospital trust. It may also inform other OPCs who plan to employ similar telemedicine solutions.

Despite some participants reporting difficulties accessing the solution, the three included components (a pre-consultation questionnaire, asynchronous digital messages, and video consultation) were generally perceived as easy to use. Perceived challenges related to the use of the telemedicine solution included not knowing who read the digital messages, challenges associated with the environment for conducting video consultations, and a perpetuated stigma by further “hiding” PLHIV from society, suggesting that aspects related to stigma have to be considered independently of the use of in-person or telemedicine care.

The participants’ perceptions of receiving current and future telemedicine care were highly dependent on the OPC’s ability to accommodate personal preferences and needs, such as type of consultations (in-person or video) and frequency of visits. In addition, the individual impacts of receiving telemedicine care were dependent on the feeling of “being seen” and preparedness of the OPC healthcare professionals during digital consultations. Nevertheless, the current telemedicine care received by the participants was perceived as a flexible and person-centred approach to care. Therefore, the telemedicine solution meets the Norwegian OPC’s aim—to enable patient follow-up beyond in-person consultations, and thereby become an extension of the OPC’s existing services. Our findings indicate that human-centred telemedicine care may support patient-centred health services and their sustainable development in the long term.

Future research is needed to understand how the use of telemedicine care affects the clinical outcomes for PLHIV. As suggested through our findings, the first step could be to develop or adjust available patient-reported outcome measures (PROMS) that can be incorporated in the pre-consultation questionnaire. The latter should be conducted in collaboration with users of telemedicine HIV care.

### Electronic supplementary material

Below is the link to the electronic supplementary material.

Additional file 1

## Data Availability

The qualitative dataset is in Norwegian and is not publicly available to protect the anonymity of the participants. Data may be available from the corresponding author on a reasonable request. The integrity and anonymity of the participants will also be maintained in that case.

## Abbreviations

COREQ: COnsolidated criteria for REporting Qualitative
HER: Electronic health record
GP: General practitioner
HIV: Human immunodeficiency virus
OPC: Outpatient clinic
PROMS: Patient reported outcomes measures
SMS: Short Message Service
