# Supplementary material for: Patients’ perceptions of use, needs, and preferences related to a telemedicine solution for HIV care in a Norwegian outpatient clinic: a qualitative study
Source: BMC Health Serv Res. 2024 Feb 15;24:209. doi: 10.1186/s12913-024-10659-z (PMC10870609; doi:10.1186/s12913-024-10659-z)
Supplement: Supplementary file 1 — Additional file 1 [file 12913_2024_10659_MOESM1_ESM.docx]

**Interview guide**

**Background information:**

• When were you born (year of birth)?

• Marital status: Are you married, in a relationship, single?

• Do you have children?

• Are you employed, a student, or doing something else?

• When were you diagnosed with HIV? (year)

• Do you feel that the HIV diagnosis affects your quality of life? If yes, in what way?

• How long have you been receiving outpatient care?

• How do you consider your competence in using digital tools?

• How familiar are you with the public website for information and access to health services for residents in Norway “helsenorge.no”?

**Follow-up before the introduction of telemedicine care:**

• How often do you usually have contact with the healthcare system due to your diagnosis?

• How would you describe the follow-up with your general practitioner (GP)?

- What works well in terms of follow-up with your GP?
- Is there anything you are not satisfied with regarding follow-up with your GP?

• Is there a clear task distribution between the GP and the outpatient clinic (OPC) related to your HIV follow-up?

• How would you define the follow-up at the OPC conducted before the use of telemedicine care?

- What worked in terms of follow-up at the OPC before use of telemedicine care?
- Is there anything you were not satisfied with regarding follow-up at the OPC before starting with telemedicine care?

• How long is your travel time to the OPC specialist (and possibly to the GP) location?

**Use of the telemedicine solution:**

• How did you experience the use of the telemedicine solution, which included a pre-consultation questionnaire, asynchronous digital messages, and video consultation?

- How did the asynchronous digital messages via helsenorge.no work?

Is there anything you are particularly satisfied or dissatisfied with regarding this solution?

Did you experience any specific challenges with using the solution?

- How did the video consultation work?

Is there anything you are particularly satisfied or dissatisfied with regarding this solution?

Did you experience any specific challenges with using the solution?

- How would you describe the use of the pre-consultation questionnaire?

Is there anything you are particularly satisfied or dissatisfied with regarding this solution?

Did you experience any specific challenges with using the solution?

**Mapping user needs and expectations related to telemedicine care:**

• What are your needs for a telemedicine care service?

• What do you think a standardized telemedicine care service should include?

- Information?
- Functionality/possibilities?
- Need for collaboration with whom?
- Layout/User interface (appearance)?
- Accessibility (platforms and time of day)

• How do you think OPC healthcare services for people with HIV should be organized?

• Do you think follow-up through telemedicine care can have an impact on your physical and mental health? If yes, in what ways?

• Do you think follow-up through telemedicine care can have an impact on your self-management and quality of life? If yes, in what ways?

• What are important aspects for you to convey regarding your follow-up through the pre-consultation questionnaire?

• Were there any questions in the pre-consultation questionnaire that you missed, did not understand, or found irrelevant?

• What do you consider as important aspects regarding the follow-up of your health condition?

**Closing question:**

• Is there anything we have not asked you that you would like to add?
